# Does the job performance of academics’ influence burnout and psychological counselling? A comparative analysis amongst high-, average-, low-, and non-performers

**DOI:** 10.1186/s12889-024-19224-z

**Published:** 2024-06-26

**Authors:** Miao Lei, Gazi Mahabubul Alam, Karima Bashir, Gui Pingping

**Affiliations:** 1 Student Affairs Division, Yancheng Teachers University, Yancheng, Jiangsu China; 2 Department of Foundation of Education, Faculty of Educational Studies, University Putra Malaysia, Serdang, Selangor Malaysia; 3 Department of Economics, East West University, Dhaka, Bangladesh; 4 Department of Education, Faculty of Education, Kebbi State University of Science and Technology, Aliero, Kebbi State Nigeria; 5 Center for Faculty Development, Sias University, Xinzheng, Henan China

**Keywords:** Job performance, Psychological counselling, Burnout, Academics, Competence, China

## Abstract

**Background:**

Extensive research has been conducted treating burnout as an independent variable and performance as a dependent variable to proffer possible solutions to burnout and job performance among academics. Despite this, the burnout crises persist and are exacerbated by the ongoing global proliferation of higher education. Acknowledging this, the current study explored whether performance may contribute to the emergence of burnout.

**Methods:**

The study’s sample population comprised 689 academics from Jiangsu province, China. Key Performance Indicator (KPI) results served to measure performance. Psychological counselling and Burnout were calculated using mental health results garnered from the universities. Data was collected on respondents' demographic characteristics and work situations. The mean scores were 0.517 (SD = 0.5) for gender and 1.586 (SD = 1.103) for age. The relationship among performance, job burnout, and psychological counselling was analysed via a cross-sectional survey deploying grouped regression.

**Results:**

Academics’ job performance was found to regulate their burnout (β = -0.058, *P* < 0.01). Higher performance of academics was significantly associated with lower job burnout and psychological counselling. Furthermore, psychological counselling significantly moderated job burnout (β = -0.012, *P* < 0.05) among academics without regulating their job performance.

**Conclusion:**

The paper supplements the discourse on job burnout and academic performance by suggesting a pre-counselling measure as a strategy to address the crises of burnout. The paper argued that the continued competence of employees should prevent burnout in Higher education and ensure better job performance.

## Introduction

Job burnout criteria and their schemata had their origins in the social and historical practices and experiences in the early to middle decades of the twentieth century. However, it was not until the mid-1970s that the factors that led to the recognition of burnout and its impacts were revealed through much psychology-based research [1, 2]. Subsequently, the strategies that had been devised for tackling the job burnout crisis were discovered or identified, which had important implications for organizations’ management and staff members’ wellbeing had to be monitored [3].

### Burnout in higher education: research gap and scope

In recent decades, the ever-increasing demands put on people who work in higher education throughout the world have intensified the pressures to deliver high-quality services to students, the wider community and internal staff members [4]. These circumstances have definitely heightened the challenges for higher education institutions to carry out effective management procedures [5]. Studies since the early 1990s have subsequently examined the correlations between job burnout issues in higher education, and other variables such as employees’ turnover [6], organizational commitment [7] and engagement with one’s work or duties [8]. To date, however, no study has yet specifically examined what sort of influence academics’ performance has on burnout – this is the focus of this research.

Studies such as those by Lei et al., Montero-Marín et al., and Watts and Robertson [9–11] explored why job burnout among academics was occurring, and what the key reasons were. What they discovered is that resource constraints, work overloads, uneven competition, inefficient and imbalanced management procedures, lack of motivation, and continuous social and family pressures are the key challenges that lead to a job burnout crisis developing. While Leiter and Maslach and Stankovic et al. [12, 13] supported earlier findings that explained the major causes of job burnout, they added that the incompetence of employees is the reason why a job burnout crisis emerged in the first place.

Later on, the findings documented in studies such as [1, 14] suggested that job training, motivation and counselling, balancing between job demands and resources, balanced distribution of tasks and working hours, resource allocation and distribution, incentives, and balancing one’s social, family or private life, all could help to address the job burnout crisis. However, the problem in higher education remains unresolved and new cases have continued to increase throughout the world.

Studies such as Fazey and Fazey, Blaskova et al. and Gillespie [15–17] further argued that the number of academic and management staff as well as students who lack the appropriate level of competence, motivation, necessary knowledge, and skills continues to grow [18]. Consequently, these situations regularly contribute to a perplexing workplace environment that works best for vested interests who put workplace pressures on the staff to carry out a myriad of tasks [19]. China presents a compelling case study, particularly in the context of its higher education system. The country has witnessed a rapid expansion of the higher education sector since 1999, changing from an elite to a mass higher education system [20]. The proliferation of higher education institutions has been stunning, growing from approximately 1,000 in 2000 to 2,756 in 2021, and the annual average growth has been 100 institutions [21]. This expansion has led to a substantial increase in university graduates from 0.83 million in 2000 to 5.054 million in 2021 [21]. Concurrently, the number of higher education academics has risen from 0.46 million in 2000 to 1.87 million in 2021 [21], meaning that China is a country with one of the largest groups of educators.

With this context in mind, it is crucial to explore the salient factors causing job burnout particularly in the huge Chinese higher education sector and identify and explain the pre-cautionary measures that could help alleviate burnout so that there is much less dependence on post-cautionary remediation measures.

### Burnout: origin, evolution, and definition

Over the past fifty years, the concept of job burnout has been the subject of substantial conceptual and methodological analysis. Job burnout is currently recognized as a multi-dimensional construct primarily associated with prolonged exposure to work-related stress [1]. Studies such as those by Maslach et al. [1, 22] defined job burnout from the psychosocial perspective, explaining it as a chronic stress-related syndrome that leads to emotional exhaustion, depersonalization or cynicism. A further feature of such burnout is the feeling of reduced or failing personal accomplishment or a sense of inefficacy.

Studies on the topic of job burnout started in the mid-1970s employing the qualitative method [22]. Basically, the qualitative studies took the phenomenological and narrative approaches, and they explained some key factors (such as work-family conflict, job autonomy, workload, years of experience, role conflicts and the state of working relationships) as the main causes of job burnout [2, 23, 24]. Then during the 1980s research tended to focus on employing various analysis models derived from quantitative methods to test the probable hypotheses where job burnout was treated as a dependent variable. Meanwhile the concerned factors were labelled as independent variables [1]. More research that continued up to the present day in the 2020s, resulted in establishing correlations/causal relationships between several factors, for instance personality, working relationships and job burnout [25–27].

**Fig. 1:**
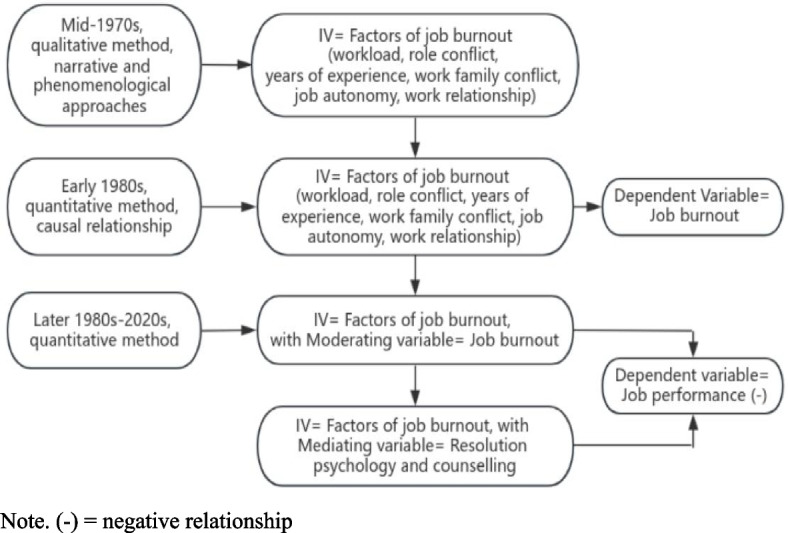
Path of job burnout research. Note. (-) = negative relationship

A recent trend has been the emergence of research concentrating on identifying the consequences of job burnout by using both qualitative and quantitative methods, as form of mixed methodology [1]. Comparative studies have been predominantly conducted where job burnout was treated as an independent variable and job performance served as the dependent variable [28]. Essentially, published studies compared between burnout and non-burnout groups and their results indicated that job burnout and job performance had a negative correlation. Despite a long history of researching job burnout through the aforementioned pathway (Fig. 1), apprehensions emerged that performance might be the primary cause for burnout.

**Fig. 2:**
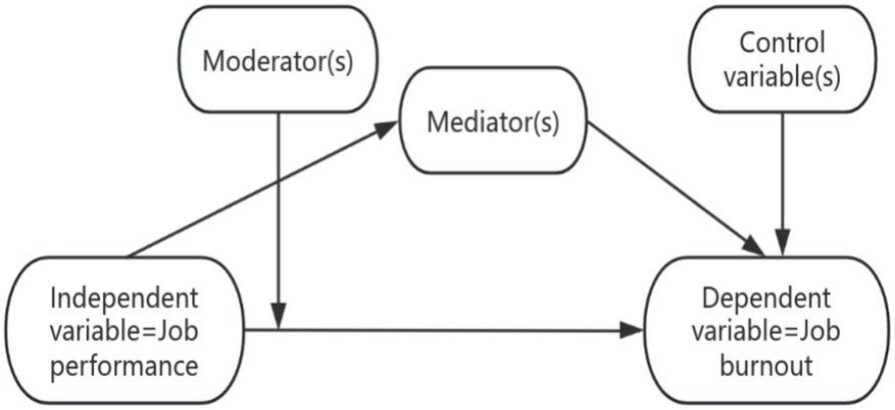
Proposed new research path

If the performance while doing one’s job triggered burnout, then earlier research on the pathways to burnout might have arrived at a completely different answer. This is despite the fact they may have reached a destination that was not fully agreed on. The ongoing research that has been done on the burnout pathway has erroneously established poor workplace functioning as a consequence of job burnout [1, 29]. Subsequently, this study treats job performance as an independent variable, while job burnout is the dependent variable. Taking this approach seeks to create a new paradigm on job burnout research, one that heralds a new pathway on this topic (Fig. 2)—travelling from the problem (job performance) to the destination (job burnout). This new research pathway will also make comparisons among four types of performing groups to establish whether job performance influences burnout while controlling for factors of burnout (gender, age, professional titles, years of employment in all groups).

### Job performance in higher education

Theoretically, job performance can be defined as an aggregate of employees’ behaviours and actions that are expected to add value, assuredness and consistency to their workplace (positive or negative). It is essentially the collective expected value of an organization that all employees are expected to help achieve what the workplace has as its objectives or goals [30]. Paudel [31] emphasized that the collective performance of a university is mainly dependent on the achievement of the academic staff. Consequently, universities measure job performance and reward actions that are aligned with teaching excellence, future career prospects and organizational policies [32, 33].

Three major methods have been identified as measuring an employee’s ability to perform his or her job. The Annual Compensation Review (ACR), also known as the annual confidential report for some countries, is one of the premier strategies implemented for measuring job performance [34]. Following ACR, the designated line managers will evaluate the employees' performance by employing descriptive analysis based on the job descriptions or duty statement [35].

However, the ACR method faced criticism for being arbitrary as factors like nepotism, prejudice, favouritism, and office politics, rather than actual job performance often influenced higher performance scores [34]. To reduce or remove such bad influences, multiple stakeholders were engaged within the ACR to eliminate these. However, it simply failed because what grew in the workplace was a lobbying, imitative and group-based culture in the workplace. The right things could be said or done but without much integrity behind them, so for this reason the ACR became an ineffective tool.

The failure of the ACR model led to the rise of the Individual Work Performance Questionnaire (IWPQ) model [36] that also later failed due to several reasons which include (1) the IWPQ model failed to identify the specific realities of workers’ performance (2) all employees do not work on the same hierarchical level, so the IWPQ model failed to distinguish between certain specified performance expectations and skills; (3) a basic theoretical test of performance may not reflect the practical reality; and what their duty statements demanded [37].

The incompatibility of both models (ACR and IWPQ) made the Key Performance Indicator (KPI) model popular over the last few decades [38]. In order to ensure a more accurate KPI, a 360-degree approach was introduced since 1990s [39]. Based on this model, the tasks and responsibilities of an employee are segmented into various micro units that reflect a range of differentiated or diverse domains. The specified tangible targets identified for each KPI unit are assigned along with the determined score for each employee based on his/her level in the hierarchy [40].

Determining the score that goes towards the KPI would normally be maintained through both qualitative and quantitative parameters, and this involves many stakeholders who work in the system [38]. Despite criticism labelling it as a “numbers game,” the KPI system has become a globally accepted model for assessing how well university academics perform [39] and China is no exception here. Hence, the score obtained on a KPI is the only official measurement method of evaluating the academics’ job performance and would be used as secondary data.

### Research aim, questions, and hypothesis development

Current research trends are highlighting job burnout as the cause of certain job performance behaviours being labelled by Lemonaki et al. [41], although Taris [29] acknowledged that job burnout greatly determines performance. Challenging this belief, it has been highlighted that staff members’ performance should ideally influence the issues of job burnout in the university which is yet to be studied scientifically since the 1970s. Hence, whether an obvious difference exists in connection to job burnout between high—and low—performing groups is a key question and answering it will contribute to further refinement on the topic of job burnout.

Keeping this view in mind, this study seeks to compare between the performance level among groups of academic staff in order to: (1) re-explore the possible reasons causing job burnout in the higher education sector; and (2) offer some pragmatic measures applicable to both before and during the job that lead to preventing or at minimizing the burnout crisis. The following questions have been devised to get to the heart of the matter in accordance with the research aim and objectives:

1. What is the influence of job performance on job burnout? and
2. What mechanism/measures can be designed to address job burnout?

In order to answer these research questions, the hypotheses devised for this study are explained below.

In the latest revision of the International Classification of Diseases (ICD-11, 2019), the World Health Organization (WHO) officially designates burnout as an occupational disease, cautioning against its application to describe experiences outside of the workplace. Sunjaya et al. [42] further argued that it could – in severe cases – lead to medical conditions or health-threatening scenarios such as cardiovascular disease, cardiovascular risk factors, and depression. Similarly, the WHO [43] recommended psychological counselling and medical services as the main strategies to treat work issues such as job burnout in many societies, but here the basic well-known principle of “prevention is better than cure” [3, 44] was not adhered to. This study intends to hypothesize that job performance fundamentally shapes job burnout by challenging the currently established hypothesis — job burnout primarily hampers good job performance:

- H_a_1: Academic job performance wields a significant negative influence on job burnout.
- Ha2: There is a significant difference in the role of psychological counselling among the four different job performance groups.

If these two hypotheses are proven to be correct, a strong case would subsequently be established where the argument is made that more competent or resilient individuals with high job performance attributes need to be recruited as a pre-cautionary measurement. Their recruitment will prevent or at least greatly minimize the job burnout crisis without having to rely on psychological counselling as a post-cautionary measure. This marks an important contribution the “prevention is better than cure” school of thinking in job-burnout research. Discussions that followed explained the foundation that motivated the authors to develop these hypotheses.

Globally, burnout research was conducted in university settings due to the rising incidents of overloaded workplace demands, coupled with a decline in the number of staff as automation took over certain functions, and the insufficient coping competencies displayed by academics [10]. Studies have argued that academics are feeling the strain of various challenging occupational demands, including having to deliver consistently high-quality teaching and supervision to students, publishing innovative research in high-impact journals, sustaining managerial and entrepreneurial skills and responsibilities to a high level, and tenure-related success [11]. When they cannot do so, this leads to the competency of academics being seriously questioned.

Statistics reveal there has been a huge surge in the worldwide number of academics from 4 million in 1980 to 13.1 million in 2018, made possible by the emergence of mass higher education in developing and developed countries [45]. However, the increase in quantity does not mean that there was a similar increase in the quality of academics, making it impossible to match the standards of elite education [11]. Furthermore, Montero-Marín et al. [10] opined that academics worldwide experience persistent job burnout due to a lack of skills or competency to meet substantial work demands. Using this perspective, this paper developed subsequent hypotheses based on our core hypothesis explained earlier in this sub-section:

- Ha3: Psychological counselling as a post-cautionary measure has a significant positive influence on job burnout.
- Ha4: Psychological counselling as a precautionary measure has a significant positive influence on job burnout.

The results of these four hypotheses can lead to establishing further results and discussions for our research questions outlined earlier. Drawing from the model and existing literature, our study employs a quantitative research method using the comparative analysis approach to explore the influence of job performance on job burnout. The following section explains the research methods, procedure and the population and sample developed for this study.

## Methods

The quantitative method, using cross-sectional survey design was employed for this research. As advocated by Creswell [46], this method is deemed most suitable to yield precise statistical results and elucidate relationships between variables, for example examining the statistical associations between academics' performance and job burnout.

### Population and sampling techniques

Jiangsu province, a major economic hub in China and ranking second nationwide is the focus of this study. According to China Statistical Yearbook [20], Jiangsu province has a total of 167 universities, and it is ranked first in the country. These universities can be categorized into three types, i.e. Double First-Class university, general undergraduate universities and vocational universities [47]. To achieve a representative sample, a multi-stage sampling approach is implemented, and this method involves identifying different strata, selecting target individuals within these strata, and then sampling from these individuals [46]. The total sample from the total academic population amounts to 5694 and this number refers to academics who are working in Jiangsu province in its 167 universities. Since the nature of universities varies, one university is sampled from each type of university in order to achieve triangulation.

Following the recommendations of The Research Advisors [48], a sample size of 689 was deemed necessary for a population of 5694 academics. Demographic characteristics of the sample are presented in Table 1. Subsequently, this sample was proportionally divided and allocated through a stratified sampling technique, making it possible to select specific respondents from each university included in the sampling process. Based on this approach, 408, 203, and 78 academics were sampled as presented in Table 2.

**Table 1:** Descriptive statistics of controlled variables

Control Variables	Number	Percentage %	Mean	SD
Gender				
Male	333	48.33	.517	.5
Female	356	51.67		
Age				
35 or below	149	21.62		
36–45	173	25.11	1.586	1.103
46–55	181	26.27		
56 or above	186	27.00		
Professional title				
Professor	89	12.92		
Associate Professor	218	31.64	1.611	.932
Lecturer	254	36.86		
Teaching assistants	128	18.58		
Years of employment				
5 or less	114	16.54		
6–10	84	12.19		
11–15	107	15.53	2.904	1.989
11–20	96	13.93		
21–25	116	16.84		
26–30	81	11.76		
31 or above	91	13.21		

*N* = 689, *SD* = Standard deviation

**Table 2:** Sampling and grouping

University type	TA	HP	AP	LP	Non-P	SA
Double First-Class university	3371	80	160	87	81	408
General undergraduate university	1680	44	87	37	35	203
Vocational university	643	17	32	15	14	78
Total	5694	141	279	139	130	689

*N* 689, *TA* Total academics, *HP.* High performance, *AP.* Average performance, *LP.* Low performance, *NP.* Non-performance, *SA* Sampled academics

The personnel departments of selected universities assisted in randomly selecting the academics involved in the study based on every third name on the staff list. These selected academics were used for all the data collection and analysis. The randomly selected samples were grouped based on the 4 categorizations of job performance (high, average, low and non-performance). This helped us not just to avoid sample bias but also provide a random visualization of the performance levels of academics.

### Data collection and procedures

The collection of data took place between January 3 and February 17, 2024, and the sources were the university archives. All the universities were approached, and the first author explained the objective of the study to the relevant authorities and what information was required from the universities. Before the visits, ethical approval such as concerns about the safety of the research was obtained from a university (REFERENCE NO: JKEUPM-2023–676) before the research commenced. Subsequently, permissions were obtained from each university. Each university approved the request and provided the researchers with a reference number (YCTU20221017). Selected universities were made aware that participation is voluntary, and they could withdraw from the study at any time.

To ensure participants’ anonymity, we assigned numerical codes sequentially, starting with the first sample from the list. Thus, we collected samples from the universities with respondents represented as 1, 2, 3, 4, etc. Therefore, all the participants’ personal information were anonymous and unknown to the researchers. Furthermore, universities were assigned with letters of the alphabet, i.e. A, B, and C. As such, the code for respondents in university A (the first university we collected information from) is A1, A2, etc., to the last respondent. For the second university, we used B1, B2, etc., and pseudonyms were implemented in all the documents as collected from the universities.

### Instruments: measures and control variables

The data collection involves demographic information, Key Performance Indicator (KPI) results, and psychological counselling data from the sampled universities which derive from the same individuals. The use of secondary data obtained from the personnel department of the sampled university is more reliable than survey responses.

#### Control variables

The demographics information: gender, age, professional title, years of employment were evaluated and included in the regression model due to their established associations with job burnout [49, 50].

#### Measurement of job performance

Key Performance Indicator (KPI) was used to measure the performance of the academics. In China, specific KPIs for measuring each competency and performance of each academic is mandated on universities, the basis for this practice being the Guiding Opinions on Deepening the Reform of the Assessment and Evaluation System for Universities Teachers [51]. According to Regulations on the Assessment of Staff in Public Institutions [52], academic performance is generally divided into four categories: high performance (excellent), average performance (qualified), low performance (basically qualified), and non-performance (unqualified). Hence, KPI results and grading will be utilized as groupings to measure the job performance of academics in this study.

The four-year KPI results of academics were compiled to highlight multi-year trajectories. The inclusion criteria require all academics to have fully participated consecutively in the KPI from 2019 to 2023. The exclusion criteria applied to academics who missed the entire KPI or missed a KPI between 2019 and 2023. In essence, academics who have retired or were absent were not included.

#### Measurement of burnout and psychological counselling

Burnout and psychological counselling data is used to calculate the burnout status and psychological counselling records of academics. The Guiding Opinions on Strengthening Psychological Health Services from [53] stipulated that all higher education institutions should offer psychological health services, psychological assessments and other related services regularly for staff members. Data on burnout and psychological counselling was directly obtained from the Mental Health Centre of this study’s sampled universities. According to the records, burnout results and consultation frequency, and psychological counselling were scored based on a scale and were utilized for this study. These forms of data delivered a visual representation of information on academics who benefit from psychological counselling yearly. The measures are presented in Table 3.

**Table 3:** Measures

Variables	Tools/Instruments	Domains
Burnout Dependent Variable	Mental Health Centre records	Non-burnout -0
		Low burnout -1
		Moderate burnout -2
		High burnout -3
Job performance Independent Variables	Key Performance Indicator (KPI)	Non-performance -0
		Low performance -1
		Average performance -2
		High performance – 3
Psychological counselling Moderating Variable	Mental Health Centre records	Non-psychological counselling – 0
		Monthly Sessions -1
		Bi-Weekly Sessions -2
		Weekly Sessions -3

### Data analysis

For the first research question (RQ1) concerning the relationship between job performance and job burnout, a multiple regression analysis was conducted using the latest data. To further investigate the relationship between job performance and job burnout and avoid errors caused by sample heterogeneity, group linear regression was employed. This served to examine the impact of job performance on job burnout among the four job performance groups (Model 1).

A performance group comparison based on burnout in 4 years was mapped out to document the results of RQ1 (Model 2). Furthermore, whether job performance affects burnout while controlling for gender, age, and years of employment was assessed. Regarding the second research question, hierarchical linear regression is first employed to assess psychological counselling in terms of: firstly, whether it significantly influences job burnout; and secondly, whether it has a moderating effect.

To ascertain the finding in the first step and to avoid errors caused by sample heterogeneity, group regression analysis was conducted among the four performance groups. This was done to determine whether psychological counselling wields a moderating effect amongst the four job performance groups (Model 1). Furthermore, a performance group comparison based on the role of psychological counselling for more than four years was presented to support the results of RQ2 (Model 2). The research questions and statistical methods are presented in Table 4.

**Table 4:** Research questions and analysis techniques

Research questions	Hypotheses	Methodology	Analysis
What is the influence of job performance on job burnout?	H_a_1: Academic job performance wields a significant negative influence on job burnout	Quantitative	Linear regression, frequency trends
	Ha2: There is a significant difference in the role of psychological counselling among the four different job performance groups		
What mechanism/measures can be designed to address job burnout?	H_a_3: Psychological counselling as a post-cautionary measure has a significant positive influence on job burnout	Quantitative	Linear regression, frequency trends
	H_a_4: Psychological counselling as a precautionary measure has a significant positive influence on job burnout		

## Results

### The influence of job performance on job burnout

Table 5 displays the results of the multiple linear regression analysis, and an inverse relationship between job performance and job burnout among academics (β = -0.058, *p* < 0.01) is noted. This finding supports H_a_1.

**Table 5:** Influence of job performance on job burnout

Variables	Overall
Gender	-.052
	(.060)
Professional title	-.011
	(.032)
Age	.001
	(.027)
Years of employment	-.022
	(.015)
Job performance	-.058***
	(.002)
Constant	5.787***
	(.169)
N	689
R-squared	.572

*N* sample size, Index in brackets = Standard error

^***^p < .01

To further confirm Ha1, a group regression analysis was executed among the four performance groups to assess how much influence performance level has on job burnout. Table 6 summarizes the findings, strongly suggesting that there is a notable negative association between job performance and job burnout across all four performance groups. Specifically, job performance demonstrates a negative effect on job burnout within each group.

**Table 6:** Influence of job performance on job burnout among 4 groups

	HP	AP	LP	Non-P
Gender	.049	.071	-.278**	-.113
	(.089)	(.062)	(.120)	(.137)
Professional titles	.017	-.031	.085	-.016
	(.045)	(.034)	(.066)	(.074)
Age	.012	-.036	.017	.123**
	(.039)	(.027)	(.057)	(.064)
Years of employment	-.007	-.011	-.058**	-.025
	(.023)	(.015)	(.030)	(.037)
Job performance	-.068***	-.150***	-.221***	-.062***
	(.015)	(.005)	(.016)	(.011)
Constant	6.675***	13.059***	16.469***	5.429***
	(1.381)	(.441)	(1.065)	(.637)
N	141	279	139	130
R-squared	.138	.742	.598	.263

*N* sample size, *HP.* High performance, *AP.* Average performance, *LP.* Low performance, *NP.* Non-performance, Index in brackets = Standard error

^***^p < .01

^**^p < .05

**Fig. 3:**
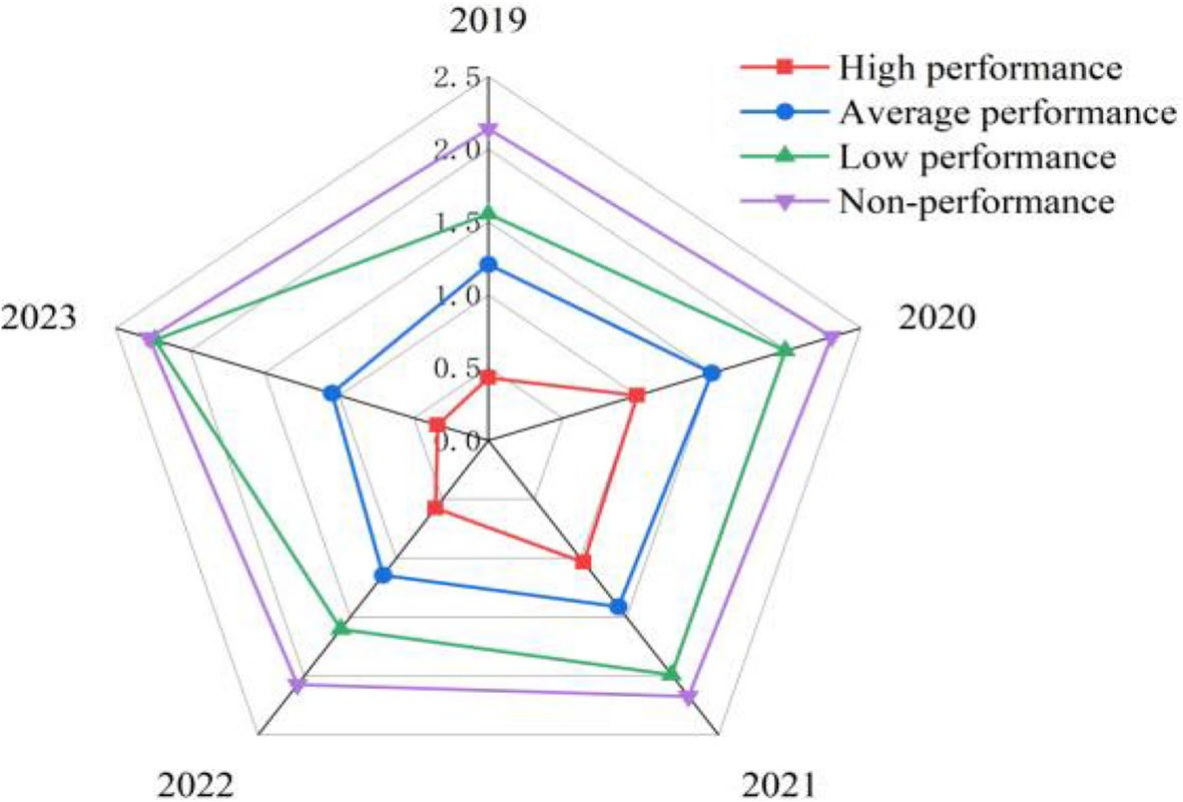
Academics Job performance group comparison (2019 to 2023)

To confirm the inverse association between job performance and burnout and to supplement the outcomes of Model 1, which suggests that performance dictates burnout, the findings from Model 2 analysis demonstrate that academics in the high-performance group consistently maintain low burnout levels. Meanwhile those in the non-performance group consistently exhibit high burnout levels, as depicted in Fig. 3.

### Moderating effect of psychological counselling

Analyzing the moderating influence of psychological counselling on academics experiencing burnout revealed that alleviating the burnout crisis can be done through psychological counselling provides substantially. In the initial block, no control variables significantly impact burnout. However, once job performance is added in the second block, it significantly contributes to negatively predicting burnout (β = -0.034, *p* < 0.01).

In the third block, it becomes evident that psychological counselling exerts a negative influence on job burnout (β = -0.160, *p* < 0.05). Upon introducing the interaction between job performance and psychological counselling in Block 4, it significantly predicts job burnout (β = -0.012, *p* < 0.05). It is strongly indicated in Table 7 that psychological counselling plays a significant negative moderating role in the relationship between job performance and job burnout among academics experiencing crises as a homogeneous group.

**Table 7:** Psychological counselling moderating the influence of job performance and job burnout

	Block 1	Block 2	Block 3	Block 4
Gender	-.100	-.097	-.097	-.078
	(.075)	(.061)	(.074)	(.060)
Professional title	-.009	-.010	-.012	-.009
	(.040)	(.033)	(.040)	(.032)
Age	.051	.020	.050	.019
	(.034)	(.028)	(.034)	(.028)
Years of employment	-.025	-.022	-.023	-.019
	(.019)	(.015)	(.019)	(.015)
Job performance		-.034***		-.027***
		(.002)		(.003)
Psychological counselling			-.160**	.693**
			(.073)	(.310)
Job performance *psychological counselling				-.012**
				(.005)
Constant	2.162***	4.491***	2.243***	4.057***
	(.115)	(.179)	(.120)	(.254)
N	451	451	451	451
R-squared	.015	.351	.025	.364

*N* sample size, Index in brackets = Standard error

^***^p < .01

^**^p < .05

Furthermore, after excluding academics who did not participate in psychological counselling, the group regression analysis, as presented in Table 8, reveals that psychological counselling exerts a moderating effect among the low (β = -0.020, *p* < 0.01) and non-performance (β = -0.036, *p* < 0.05) groups. However, there is no moderating influence in the high (β = -0.051, *p* > 0.05) and average (β = 0.006, *p* > 0.05) performance groups. Thus, the moderating influence of psychological counselling on job burnout differs across the high, average, low, and non-performance groups. This finding suggests that H_a_2 is not substantiated.

**Table 8:** Psychological counselling moderating the influence of job performance groups and job burnout

	HP	AP	LP	Non-P
Gender	.028	.102	.027	-.122
	(.089)	(.064)	(.027)	(.115)
Professional title	.022	-.020	-.006	-.095
	(.052)	(.034)	(.014)	(.064)
Age	-.024	-.008	.027**	-.041
	(.051)	(.029)	(.012)	(.063)
Years of employment	-.014	-.020	-.003	-.033
	(.026)	(.015)	(.006)	(.034)
Job performance	-.020***	-.068***	-.243***	-.079***
	(.031)	(.007)	(.003)	(.008)
Psychological counselling	1.202	.088	-.172***	-.720**
	(1.397)	(.131)	(.044)	(.351)
Job performance *psychological counselling	-.051	.006	-.020***	-.036**
	(.058)	(.015)	(.007)	(.017)
Constant	-2.621**	6.877***	17.823***	7.773***
	(3.606)	(.570)	(.226)	(.674)
N	26	84	73	48
R-squared	.240	.569	.988	.743

*N* Sample size, *HP.* High performance, *AP.* Average performance, *LP.* Low performance, *NP.* Non-performance, Index in brackets = Standard error

^***^p < .01

^**^p < .05

**Fig. 4:**
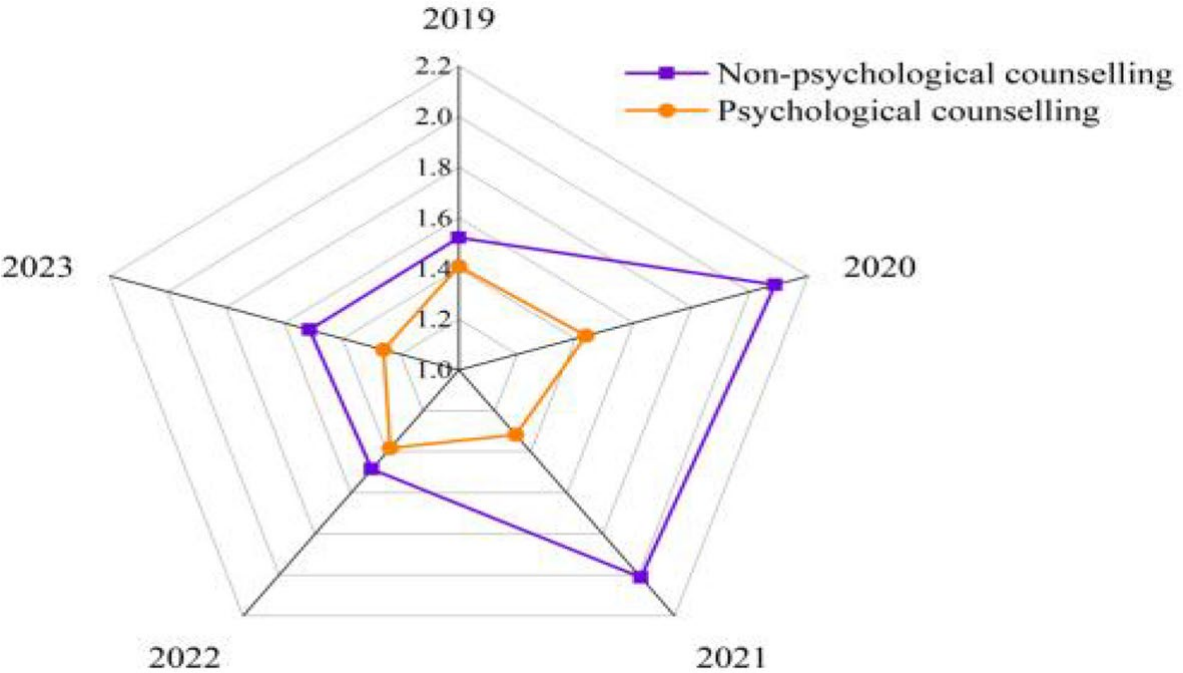
Comparing the role of psychological counselling on job burnout among four groups (2019 to 2023)

To further understand the influence of psychological counselling on burnout among the four performance groups, longitudinal data analysis was conducted on the groups receiving counselling over four years. Illustrated in Fig. 4, the trend revealed that from 2019 to 2023, academics participating in psychological counselling exhibited lower burnout levels compared to those who do not participate in counselling.

**Fig. 5:**
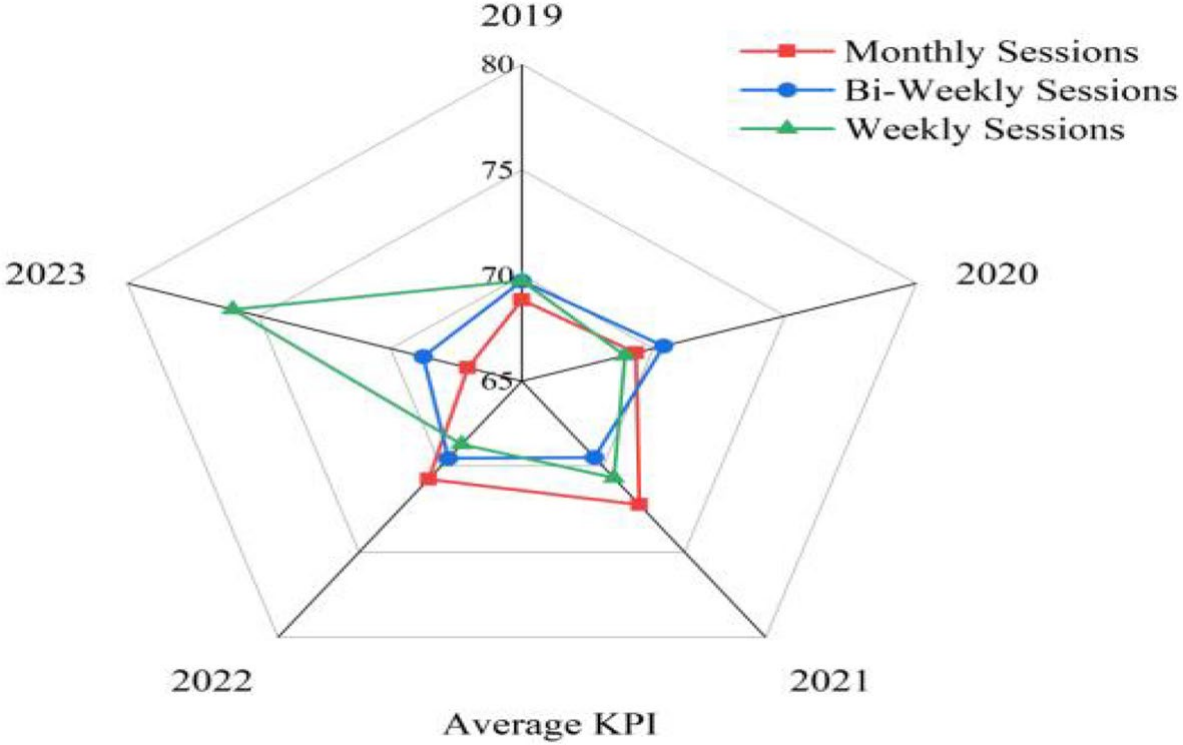
Comparison of academics KPI performance based on their frequency of psychological counselling (2019 to 2023)

Similarly, Fig. 5 shows that regardless of the frequency of psychological counselling and interventions, the KPI scores or performance of academics remain consistent. Thus, these results suggest that while psychological counselling helps to reduce burnout among academics, supporting Ha3, it does not positively affect their performance.

## Discussion

### Job-performance vs job burnout

Studies by Lei et al. and LePine et al. [9, 54] explained that academics who strive and function well would endeavour to overcome various stressors in the workplace and therefore avoid burnout. Conversely, those who struggle to perform well in their jobs will eventually suffer burnout due to their inability to cope with the demands made on them [12]. Similar to this research [12], our study found that job performance remains a significant factor influencing job burnout, even after controlling for variables such as gender, age, marital status and others.

Additionally, in the process of improving performance, academics become more expert in their tasks and what they accomplish, which has can create individual benefits (for example, promotion or job recognition) from the organization as reported by Weng et al. [55]. This finding also supports Taris and Schaufeli [56] and provides further evidence that the job resources (job performance) acquired can alleviate or reduce job burnout also expands the conservation of job demands resources model [2].

This study contributes to the research on job burnout scenarios by explaining the relationship that exists between job performance and job burnout amongst academics, resulting into valuable practical outcomes. By expanding job burnout research and backing a different perception on this issue, this study makes valid and substantial theoretical contributions. While most studies typically consider minimizing employee burnout to improve their performance [57, 58], this study proposes a new methodological path to exploring the relationship between job performance and burnout.

### The role of psychological counselling in treating burnout: post-cautionary measurement vs pre-cautionary measurement

As one of the post-measurements, psychological counselling has proven its effectiveness in reducing job burnout. This proves that H_a_3 is valid. The results are largely consistent with prior research on using psychological counselling to alleviate job burnout in other contexts [44, 59]. As well, the findings offer empirical evidence that psychological counselling has a moderating effect in both low and non-performance groups.

However, psychological counselling does not demonstrate a moderating effect among high and average performance groups. This stems from the conclusion drawn regarding the first research question (RQ1), suggesting that academics who perform poorly are more likely to experience burnout and consequently seek help from counselling services. Conversely, those who have high performance levels typically experience less burnout. Therefore, the necessity for counselling intervention is negated. Hence, the role of psychological counselling as a post-measure to alleviate burnout remains pronounced.

Furthermore, the observed pattern on the relationship between job performance, psychological counselling and job burnout may indicate that individuals experiencing burnout may engage in a cyclical process, attempting to reduce burnout through psychological counselling. However, it should be noted that although psychological counselling may mitigate burnout supporting [44, 60] findings, it does not necessarily enhance performance as suggested by [61]. Hence, the academics may find themselves trapped in a cycle in which poor performance may experiencer recurring burnout and scenarios where stress is all-pervading. Relying on post-measurement for burnout treatment may not be sufficient for permanent resolution [62, 63] and the significance of pre-measurements, as elucidated in H_a_4 warrants in-depth exploration.

In this context, the confirmation of the “prevention is better than cure” principle within job burnout research means that H_a_4 can be supported as opined by [64, 65]. However, the relevance of academic recruitment has been either ignored or underestimated in the contemporary scholarly debate on higher education and job burnout [66]. Hence, to mitigate job burnout amongst academics and enhance their performance, a proactive recruitment system as a pre-measurement strategy should be implemented to avert any signs of a job burnout crisis. In effect, those who are recruited should be able to avoid signs of burnout.

Since counselling is not ideally meant to improve workplace performance, counselling does not automatically lead to competent, re-energized, motivated, passionate and competent staff. Conversely, a decline in performance may contribute to the persistence of burnout. In light of these findings, it is recommended that a comprehensive assessment of all candidates’ skills and attributes be incorporated into human resources recruitment processes. This means examining potential staff members’ mental, academic and psychological domains.

Furthermore, looking at the modern era which has witnessed a dramatic expansion in higher education, many individuals especially in developing countries wrongly assume that working as academics translate into being able to work less and having less or minimal commitment to their workplace [67]. This assumption should be changed before and after employment commences. Hence, university recruitment systems should incorporate psychological counselling tests to back up competency skills if these institutions want to employ competent, passionate, and self-motived individuals who are ready to work as academics.

Applicants should be comprehensively assessed, ensuring their ability to work very hard and diligently, and establishing their psychological fitness by evaluating motivations, passions and determinations relevant to the academic role. After the recruitment of right personnel, arrangements should be made for continuous professional development [68] (both academic and psychological) so that they have the ability to cope with ongoing changes in the higher education system and especially with rapid advances in technology [69]. Moreover, the university management teams should improve training programs that can enhance staff members’ job performance and expertise by addressing where their skills are lacking and enhancing their mental health [70].

### Limitations and future studies

Although this study employed a different path to explore whether job performance influences burnout, it has some limitations. Firstly, this study proposed psychological tests should be included during the recruitment drives, but it did not specify the psychological parameters to be used in these tests. Secondly, the generalizability of the findings may be constrained because the samples originating from only one province in China. It is essential to recognize the diverse nature of workplaces, cultural practices, geographies, industries, traditions, and contexts given that China is not a homogenous country. To ensure the universality of the findings, future research should include a more diverse and larger sample.

## Conclusions

The study expanded on the job burnout research to present findings that establish the influence of job performance on burnout. Indicated by the results is that performance is one of the causes of job burnout in university settings. The study also discovered that psychological counselling moderates the relationship between job performance and burnout. Although the findings confirmed that burnout can be resolved through psychological counselling, this does not mean that a non-performing group can be turned into a performing group. Therefore, to prevent job burnout, universities should refine and focus their recruitment strategies. A panel of psychologists should be included to assess the cognitive and emotional fitness of candidates, and not overly relying on their academic skills and medical screenings.

## Data Availability

The data that support the findings of this study are available on request from the first author (ML) upon reasonable request.

## Abbreviations

ACR: Annual Compensation Review
IWPQ: Individual Work Performance Questionnaire
RQ: Research Question
WHO: World Health Organization
